# Economic hardship and perpetration of intimate partner violence by young men in South Africa during the COVID-19 pandemic (2021–2022): a cross-sectional study

**DOI:** 10.1186/s40621-024-00483-8

**Published:** 2024-01-16

**Authors:** Campion Zharima, Rishav Singh, Kalysha Closson, Mags Beksinska, Bongiwe Zulu, Julie Jesson, Tatiana Pakhomova, Erica Dong, Janan Dietrich, Angela Kaida, C. Andrew Basham

**Affiliations:** 1 Perinatal HIV Research Unit (PHRU), Faculty of Health Sciences, University of the Witwatersrand, Johannesburg, South Africa; 2 Centre for Health Policy, School of Public Health, University of the Witwatersrand, Johannesburg, South Africa; 3 Vaccine Evaluation Centre, BC Children’s Hospital and Research Institute, Vancouver, BC Canada; 4 Center On Gender Equity and Health, School of Medicine, University of California San Diego, La Jolla, CA USA; 5 Faculty of Health Sciences, Simon Fraser University, Blusson Hall Rm 10522, 8888 University Drive, Burnaby, BC V5A1S6 Canada; 6 MRU (MatCH Research Unit), Department of Obstetrics and Gynaecology, Faculty of Health Sciences, University of the Witwatersrand, Durban, South Africa; 7 Center for Epidemiology and Research in POPulation Health (CERPOP), Inserm, Université de Toulouse, Université Paul Sabatier, Toulouse, France; 8 Health Systems Research Unit, South African Medical Research Council, Bellville, South Africa; 9 African Social Sciences Unit of Research and Evaluation (ASSURE), A Division of the Wits Health Consortium, Johannesburg, South Africa; 10 Women’s Health Research Institute, Vancouver, BC Canada; 11 Division of Pharmacoepidemiology and Pharmacoeconomics, Department of Medicine, Brigham and Women’s Hospital/Harvard Medical School, Boston, MA USA

**Keywords:** Financial Stress, Intimate Partner Violence, Adolescents, Masculinity, SARS-CoV-2, South Africa, Pandemics, Young Adults, Food Insecurity, Surveys and Questionnaires, Cross-Sectional Studies, Propensity Score, Logistic Models

## Abstract

**Background:**

Economic hardship is a potential trigger for intimate partner violence (IPV) perpetration. While higher IPV rates have been reported in low-income regions, few African studies have focused on IPV being triggered by economic hardship among young men during the COVID-19 pandemic. We therefore estimated economic hardship’s effect on IPV perpetration by young men in eThekwini District, South Africa, during the COVID-19 pandemic.

**Methods:**

A cross-sectional survey of COVID-19 pandemic experiences was conducted among youth aged 16–24 years through an anonymous self-administered questionnaire, including questions about economic hardship (increased difficulty accessing food or decreased income) and IPV perpetration. A prespecified statistical analysis plan with a directed acyclic graph of assumed exposure, outcome, and confounder relationships guided our analyses. We measured association of economic hardship and IPV perpetration through odds ratios (ORs) computed from a multivariable logistic regressions adjusted for measured confounders. Secondary outcomes of physical and sexual IPV perpetration were analyzed separately using the same specifications. Propensity score matching weights (PS-MW) were used in sensitivity analyses. Analysis code repository: https://github.com/CAndrewBasham/Economic_Hardship_IPV_perpetration/

**Results:**

Among 592 participants, 12.5% reported perpetrating IPV, 67.6% of whom reported economic hardship, compared with 45.6% of those not reporting IPV perpetration (crude OR = 2.49). Median age was 22 years (interquartile range 20–24). Most (80%) were in a relationship and living together. Three quarters identified as Black, 92.1% were heterosexual, and half had monthly household income < R1600. We estimated an effect of economic hardship on the odds of perpetrating IPV as OR = 1.83 (CI 0.98–3.47) for IPV perpetration overall, OR = 6.99 (CI 1.85–36.59) for sexual IPV perpetration, and OR = 1.34 (CI 0.69–2.63) for physical IPV perpetration. PS-MW-weighted ORs for IPV perpetration by economic hardship were 1.57 (overall), 4.45 (sexual), and 1.26 (physical).

**Conclusion:**

We estimated 83% higher odds of self-reported IPV perpetration by self-reported economic hardship among young South African men during the COVID-19 pandemic. The odds of sexual IPV perpetration were The seven-times higher by economic hardship, although with limited precision. Among young men in South Africa, economic hardship during COVID-19 was associated with IPV perpetration by men. Our findings warrant culturally relevant and youth-oriented interventions among young men to reduce the likelihood of IPV perpetration should they experience economic hardship. Further research into possible causal mechanisms between economic hardship and IPV perpetration could inform public health measures in future pandemic emergencies.

## Background

Intimate partner violence (IPV) is any behavior within an intimate relationship that leads to psychological, physical, or sexual harm to those in the relationship (World Health Organization 2012). A global cohort study using data from 2000 to 2018 estimated that 27% of women aged 15–49 years have experienced either physical or sexual IPV (Sardinha et al. 2022). First IPV experiences often start in adolescence (Devries et al. 2013). In low-income countries, women generally report higher lifetime and past year experiences of IPV (Sardinha et al. 2022). Perpetrators are predominantly men (Fulu et al. 2013). IPV perpetration has been associated with lower socioeconomic status, alcohol misuse, and childhood trauma (Fulu et al. 2013; Keilholtz et al. 2023; Shai et al. 2019). The COVID-19 pandemic added economic hardship (Mahlangu et al. 2022; Nyashanu et al. 2020) and increases in IPV were observed globally (Mahlangu et al. 2022; Nyashanu et al. 2020; Peitzmeier et al. 2022).

Although many people faced a loss of income, food insecurity is also considered a form of economic hardship (Ngarava 2022) and a known IPV catalyst (Jewkes et al. 2011; Hatcher et al. 2022, 2019). Besides leading to hunger, food insecurity fosters anxiety surrounding one’s access to food and may affect their ability to find food in acceptable ways (Hatcher et al. 2019). Both income loss and food insecurity negatively impact the mental well-being of young men (Lund et al. 2010; Haag et al. 2022). A study that explored COVID-19 specific risk markers for IPV perpetration showed anxiety, loneliness, fear, perceived stress and substance abuse to be contributing factors potentially (Spencer et al. 2022). The prevalence of these risk markers has increased since the beginning of the pandemic (Spencer et al. 2021), contributing to the rising IPV globally (Ansah et al. 2023).

The concept of hegemonic masculinity, referring to cultural constructions of men’s higher social status and power over women, has informed theoretical understandings of IPV perpetration causal mechanisms since the 1980s and remains a subject of ongoing research and dialogue (Connell 1987; Connell and Messerschmidt 2005). Hegemonic masculinity varies from place to place and time to time and is one of many forms of masculinity (Connell and Messerschmidt 2005). While these masculinities can vary with context and time, hegemonic masculinity is posited as a goal of men. Facing poverty or economic hardship may threaten some men’s perception of achieved hegemonic masculinity (Nyashanu et al. 2020; Gittings et al. 2021). Within the gender role strain paradigm, young men who are unable to achieve hegemonic forms of masculinity may face gender role strain, which in turn may prompt construction of either more subordinate or violent masculine identities. An increase in gender role strain among men has been associated with IPV, alcohol abuse, poor mental health and poor relationship power dynamics (Yang et al. 2019; Mesler et al. 2022; Closson et al. 2020). As a result of gender role strain, caused by economic stress that led to these additional factors, relationships may have experienced heightened tensions as young adults deal with new stressors.

A study of economic stress and lockdown effects on IPV perpetration in Spain found a 23.38% increase in IPV incidence, driven by increased sexual IPV and psychological IPV and not physical IPV (Arenas-Arroyo et al. 2021). Often conflated, lockdown and economic stress had independent effects on IPV in that study, with economic stress having double the effect of lockdown on overall IPV incidence (Arenas-Arroyo et al. 2021). Lockdown without economic stress significantly affected psychological IPV after adjusting for age, demographics, and employment status, but not physical or sexual IPV (Arenas-Arroyo et al. 2021). This is not surprising as lockdown measures share common traits with psychological IPV, such as social isolation and monitoring daily activities and movement (Gelder et al. 2020). A study of IPV during COVID-19, among pregnant women, using a longitudinal design measuring economic hardship weekly estimated a within-person effect of increasing the odds of IPV by 28% in the same week (Cochran et al. 2023).

A recent meta-analysis of life stressors as risk factors for IPV summarized evidence that unemployment can be a trigger for IPV perpetration by men (Keilholtz et al. 2023). However, perceptions of financial stress, rather than objective measures of financial resources (income-to-needs ratio) may be predictive of IPV perpetration, implying that financial management interventions could be more effective than interventions to increase financial resources, in preventing IPV perpetration (Schwab-Reese et al. 2016).

Economic hardship created by COVID-19 may have intensified challenges to young men’s perceived socioeconomic status and perceived masculinity, leading to heightening gender role strain in relationships and, consequently, the risk of IPV perpetration. In this study, we aimed to estimate the effect of economic hardship on the perpetration of IPV by young men living in South Africa during the COVID-19 pandemic. We hypothesized that economic hardship during the COVID-19 pandemic would be associated with perpetration of IPV. We further hypothesized that socioeconomic status and relationship status both modified the effect of economic hardship on IPV perpetration risk.

## Methods

### Study population, data collection, and analytic sample

Our study population included men aged 16–24 years living in the eThekwini district of South Africa. The eThekwini Metropolitan Municipality is the third largest city in the country and is a major tourist destination due to its climate and sea point location (Maharaj et al. 2008). Participation required the ability to read and write in English and/or isiZulu, with access to a mobile phone, tablet, or computer with internet service available.

A multi-pronged recruitment strategy was used as the AYAZAZI RIGHTS (Rapid Investigation of Gendered Health outcomes in the Time of SARS-CoV-2) survey recruited participants from a pool of over 6000 adolescents. First, the recruitment team reconnected with previous participants from MatCH (Maternal, Adolescent and Child Health) Research Unit studies in Durban who had agreed to be part of future research. Second, collaboration with community-based and youth-led organizations, particularly the Adolescent Community Advisory Board. Other strategies included distributing flyers in areas frequented by youth, online advertising, sending emails, and reaching out to schools and universities. Eighty percent of recruitment were from a friend (38%), flyer (24%), or school/university (18%).

Phone and device connectivity were provided free to complete the survey through the Moya messenger app. Data were collected anonymously online through self-administered questionnaires from the 21st of December 2021 to the 31st of May 2022. The survey comprised forty-four questions across four sections: demographics and socioeconomic status, COVID-19 experiences; sexual and reproductive health; and mental health in the context of the COVID-19 pandemic.

**Fig. 1:**
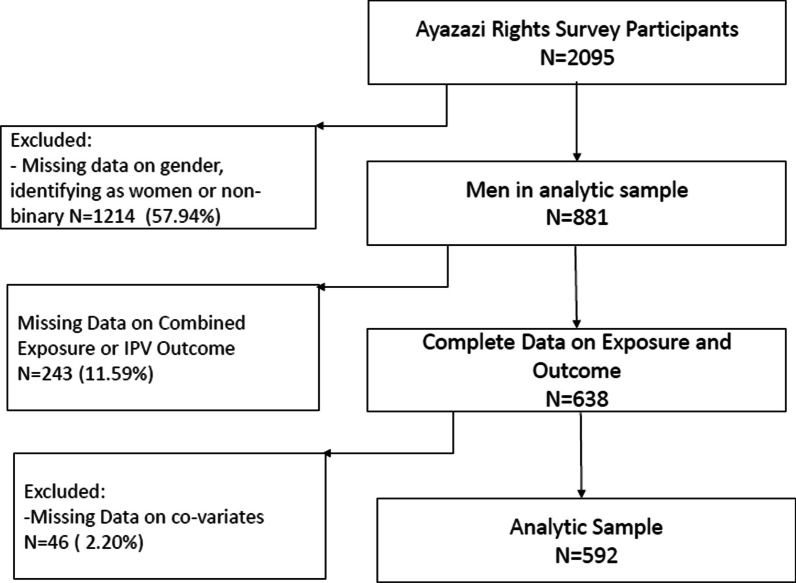
Flow chart for analytic sample of young men in eThekwini District, South Africa

Our base sample (*N* = 2095) included participants who completed the survey in > 4.5 min (Fig. 1). Most participants in the base sample identified as women (52.36%) followed by men (42.05%). Additionally, 4.30% identified as non-binary and 1.29% were missing data on gender identity. Overall, 83.60% were heterosexual. A majority were aged 20–24 (69.50%) with the remainder aged 16–19. At the time of survey completion, 73.70% reported being in a relationship.

To create our analytic sample, we excluded anyone who did not identify as a man, had missing data on exposure or outcome, or were missing a value for any of the covariates (Fig. 1). After applying exclusion criteria, our analytic sample (*N* = 592) included 12.5% (n=74) men who reported perpetrating IPV either physically or sexually (Table 1). The median age of participants was 22 years (IQR 20–24). Nearly three quarters of participants identified as Black (*n* = 435, 73.5%). Forty-seven participants (7.9%) identified as non-heterosexual (gay, bisexual, queer, questioning, or asexual). A total of 236 participants (45.6%) reported experiencing economic hardship, and among them, the majority (*n* = 50; 67.6%) reported perpetrating IPV during the pandemic, yielding a crude OR of 2.49 (95% CI 1.50–2.43) (Table 1). Men perpetrating IPV were more likely to be in the lowest or highest income categories, less likely to be employed or in school, more prone to experiencing anxiety, worry, distress, or inability to cope during the COVID-19 pandemic, not disclose their HIV status and were more likely to consume alcohol ≥ 1/week (Table 1).

**Table 1:** Analytic sample of men ages 16–24 years in the eThekwini District, South Africa, during the COVID-19 pandemic, 2021-2022: bivariable logistic regressions of IPV perpetration on potential confounders

Characteristic, *N* (%) unless noted	IPV perpetration		OR (95% CI)
	Yes	No	
Overall^a^	74 (12.5)	518 (87.5)	N/A
Economic Hardship	50 (67.6)	236 (45.6)	2.49 (1.50–2.43)
Age, *years* mean (SD)	22.27 (1.82)	21.67 (2.40)	1.13 (1.00–1.27)
Race, *Black*	57 (77.0)	378 (73.0)	1.24 (0.70–2.21)
School or employment	49 (66.2)	386 (74.5)	0.67 (0.40–1.13)
Sexual orientation			
Heterosexual	64 (86.5)	481 (92.9)	Ref
GBQQA	10 (13.5)	37 (7.1)	2.03 (0.96–4.28)
Household income level			
I do not get an income	15 (20.3)	54 (10.4)	Ref
R1-R800	14 (18.9)	132 (25.5)	0.38 (0.17–0.84)
R801-R1600	8 (10.8)	106 (20.5)	0.27 (0.11–0.68)
R1601-R3200	11 (14.9)	92 (17.8)	0.43 (0.18–1.00)
R3201+	26 (35.1)	134 (25.9)	0.70 (0.34–1.42)
Relationship status			
No partner	19 (25.7)	52 (10.0)	Ref
Partner and living together	40 (54.1)	436 (84.2)	1.37 (0.61–3.08)
Partner and not living together	15 (20.3)	30 (5.8)	0.25 (0.14–0.47)
Has one or more children	45 (60.8)	191 (36.9)	2.66 (1.61–4.38)
Household composition			
Children in household, *mean (SD)*	2.69 (1.22)	2.20 (1.35)	1.30 (1.09–1.56)
Adults in household, *mean (SD)*	3.24 (1.52)	3.70 (1.45)	0.80 (0.67–0.95)
Seniors in household, *mean (SD)*	1.31 (0.81)	0.95 (1.19)	1.21 (0.99–1.49)
HIV status			
Negative	22 (29.7)	285 (55.0)	Ref
Positive	5 (6.8)	17 (3.3)	3.81 (1.28–11.30)
Unknown or prefer not to answer	47 (63.5)	216 (41.7)	2.82 (1.65–4.82)
Alcohol use frequency			
Never	26 (35.1)	177 (34.2)	Ref
Once a month or less	8 (10.8)	164 (31.7)	0.33 (0.15–0.75)
Once or more per week	40 (54.1)	177 (34.2)	1.54 (0.90–2.63)
Anxious during COVID-19 pandemic	32 (43.2)	206 (39.8)	1.15 (0.71–2.89)
Worried during COVID-19 pandemic	47 (63.5)	289 (55.8)	1.38 (0.83–2.28)
Upset during COVID-19 pandemic	34 (45.9)	130 (25.1)	2.54 (1.54–4.18)
Unable to cope during COVID-19 pandemic	29 (39.2)	107 (20.7)	2.48 (1.48–4.13)

Data source: AYAZAZI RIGHTS survey focusing on sexual and reproductive health among adolescents in South Africa.

*AYAZAZI *Understanding HIV Risk Among Youth in South Africa, *CI* confidence interval, *GBQQA* gay, bisexual, queer, questioning, or asexual, *HIV* human immunodeficiency virus, *IPV* intimate partner violence, *SD* standard deviation, *RIGHTS *Rapid Investigation of Gendered Health outcomes in the Time of SARS-CoV-2

^a^Column percentage

### Outcome: IPV perpetration

The primary outcome of IPV perpetration included either physical IPV or sexual IPV, defined as a binary indicator variable (0 = no IPV perpetration reported, 1 = IPV perpetration reported). Physical IPV perpetration was measured by the survey question “since the start of the COVID-19 pandemic, have you hit, kicked, thrown things, or done anything else to physically hurt your partner?” (Deitch-Stackhouse et al. 2015). Sexual IPV perpetration was measured with the survey question “since the start of the COVID-19 pandemic, have you forced your partner to have sex or anything sexual when they didn’t want?” (Deitch-Stackhouse et al. 2015). To assess if a particular IPV subtype might drive the hypothesized relationship between any economic hardship and IPV perpetration, the physical and sexual IPV variables were also analyzed separately as secondary outcomes.

### Exposure: experiencing economic hardship

Economic hardship was self-reported as either food insecurity or a decrease in income since the onset of the pandemic. We measured exposure to economic hardship through the following questions: “[d]id your access to sufficient, quality food change?", with responses indicating it was more difficult, had not changed, was easier, or declined. Responses indicating increased difficulty accessing food was considered exposure to food insecurity. The second exposure variable used to measure economic hardship assessed income decrease by asking participants, "Has your income changed from before the COVID-19 pandemic?". Responses reporting that income decreased “slightly” or “a lot” were classified as exposed, and those reporting that their income increased “a lot”, “slightly”, or was “unchanged” were classified as unexposed to economic hardship unless exposed to food insecurity.

### Covariates

**Fig. 2:**
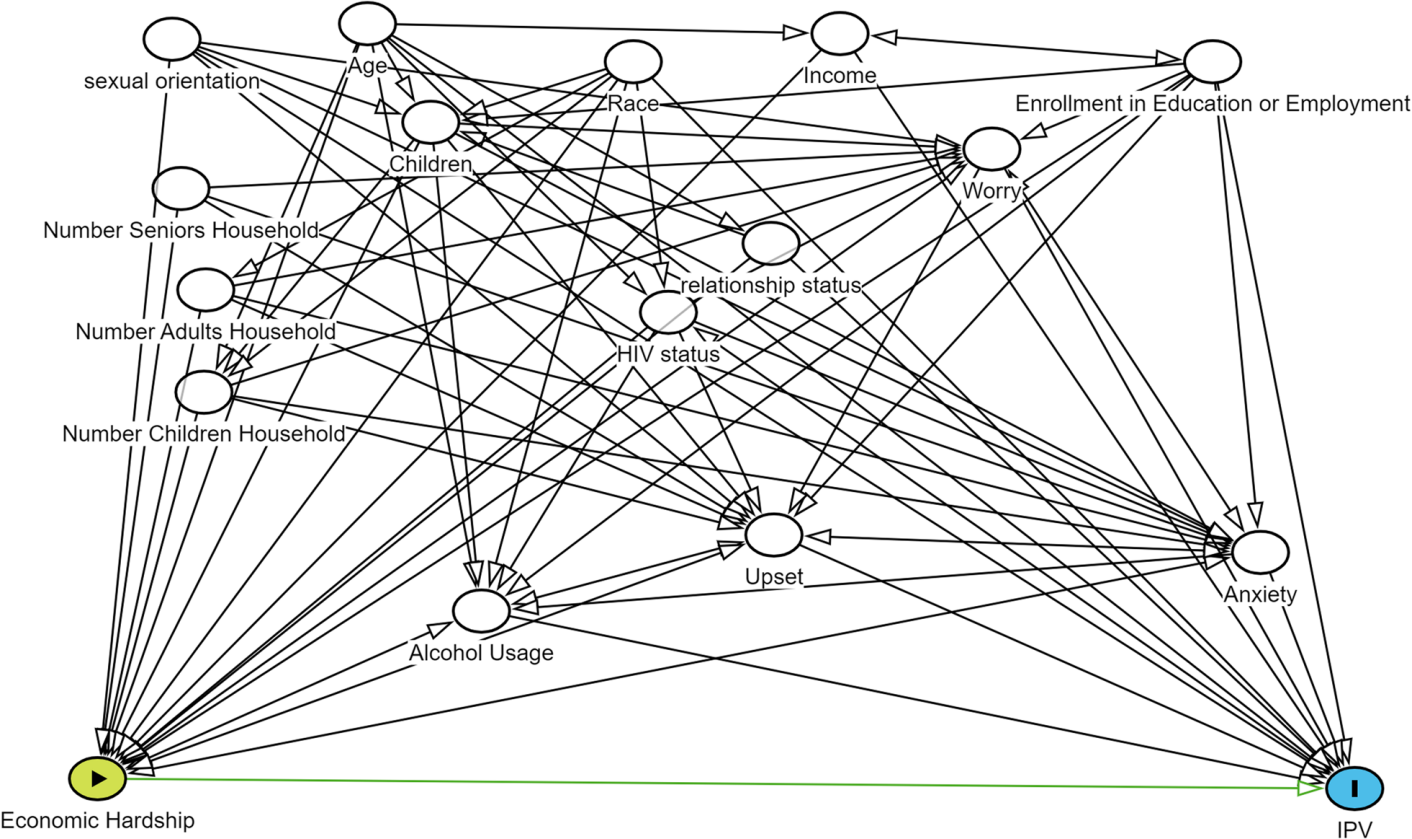
Directed acyclic graph of the effect of economic hardship on intimate partner violence perpetration

We prespecified covariates that might confound the relationship between economic hardship and IPV perpetration based on literature, content knowledge, and the modified disjunctive cause criterion (VanderWeele 2019; Gibbs et al. 2023; Acevedo et al. 2013). We graphed the assumed relationships of study variables in a directed acyclic graph (DAG) to identify our causal model (Fig. 2) and included it in a SAP developed a priori. Covariates are presented in Table 1, which is stratified by the outcome, with bivariable logistic regressions to estimate the association of each covariate with IPV perpetration.

### Statistical analysis

Self-reported perpetration of IPV was regressed on economic hardship in the primary analysis using the binomial logistic model. In secondary analyses, the effect of economic hardship on the physical IPV perpetration and sexual IPV perpetration was estimated separately using the same analytic sample and modeling strategy. The effect of economic hardship on perpetration of IPV was estimated using the odds ratio (OR) after adjusting for the covariates. In secondary analyses, the effect of economic hardship on physical IPV perpetration and sexual IPV perpetration was estimated separately using the same analytic sample and modeling strategy.

We aimed to test whether the effect of economic hardship on IPV perpetration differed by income or relationship status (Hatcher et al. 2022). We hypothesized that the effect of economic hardship on IPV perpetration during the COVID-19 pandemic lockdown was greater among people in a relationship and living together than those in relationship and not living together or who were single. Income categories of the sample were used to stratify the estimated effect of economic hardship on IPV perpetration (the OR) by socioeconomic status, which was hypothesized to be stronger among lower income groups.

To assess robustness to potential imbalance in covariates between economic hardship groups in our main analysis, we conducted a propensity score (PS) matching weight (MW) sensitivity analysis (Li and Greene 2013). We included the same covariates in a the PS model to estimate probability of economic hardship using logistic regression. We then calculated the PS-MWs, which were used to weight outcome logistic regressions of IPV perpetration on economic hardship. The resulting PS-MW weighted OR was used to estimate the causal effect of economic hardship on IPV perpetration fo primary and secondary outcomes, after balancing covariates.

Clinical and public health significance of economic hardship's effect on the risk of IPV perpetration among young men—quantified by the magnitude and precision of point estimates and confidence intervals for ORs—guided our interpretation of the results, rather than p-values and null hypothesis significance testing (Greenland et al. 2016). Only the OR for economic hardship was reported from our adjusted analyses to avoid potential for "table fallacy", whereby mutually-adjusted covariate ORs, if presented in a results table, may be inappropriately interpreted as estimates of their causal effect on the outcome (Westreich and Greenland 2013).

**Table 2:** Estimated effect of economic hardship on self-reported IPV perpetration among men ages 16–24 years in the eThekwini District of South Africa during the COVID-19 pandemic: multivariable logistic regressions

Analysis	*N*	Events	OR	95% CI
*Overall IPV Perpetration*				
Covariate-adjusted (main)	592	74	1.83	0.98–3.47
PS matching weights (sensitivity)	592	74	1.57	0.85–2.92
*Physical IPV perpetration*				
Covariate-adjusted (main)	592	57	1.34	0.69–2.63
PS matching weights (sensitivity)	592	57	1.26	0.65–2.46
*Sexual IPV perpetration*				
Covariate-adjusted (main)	592	25	6.99	1.85–36.59
PS matching weights (sensitivity)	592	25	4.45	1.16–17.13

Data source: AYAZAZI RIGHTS survey focusing on sexual and reproductive health among adolescents in South Africa.

Both covariate-adjusted and PS matching weights analyses included age, race, relationship status, sexual orientation, frequency of alcohol consumption, having children, being in school or employed, HIV status, average monthly household income, number of adults in household, number of children in household, number of seniors in household, not able to cope during COVID-19, being upset during COVID-19, anxiety during COVID-19, and worrying during COVID-19

AYAZAZI in South Africa, *CI *confidence interval, *eEvents* number of participants reporting IPV perpetration, *IPV* intimate partner violence, *N* number of participants included in analysis, *OR* odds ratio, *PS* propensity score, *RIGHTS *Rapid Investigation of Gendered Health outcomes in the Time of SARS-CoV-2

## Results

We estimated 83% higher odds of self-reported IPV perpetration, on average, among young men self-reporting economic hardship, in comparison with those who did not, after covariate adjustment (Table 2). In secondary analyses, economic hardship was estimated to have a strong effect on the odds of sexual IPV perpetration (sevenfold increase), although very imprecisely (95% CI 0.85–36.59). Economic hardship had a weaker effect estimate for physical IPV perpetration, although with greater precision (CI 0.69–2.63) due to larger number of self-reported IPV events (Table 2).

In effect measure modification analyses, a stronger association between economic hardship and perpetrating IPV in young men was estimated among those who were not in a relationship (OR = 5.18), than was estimated among young men in a relationship and living together (OR = 0.64), or in a relationship but not living together (OR = 1.73). However, confidence intervals were imprecise and overlapping, spanning 0.16–22.50 across outcome measures (Table 3). We did not observe effect modification by income group, with the largest OR in the middle-income group (OR = 2.17), with very wide confidence intervals.

**Table 3:** Effect modification in relationship of economic hardship with self-reported IPV perpetration in men ages 16–24 years in the eThekwini District, South Africa, during the COVID-19 pandemic: multivariable logistic regressions

Effect modification analysis	*N*	Events	OR	95% CI
*Relationship status*				
Not in a relationship	71	19	5.30	1.25–22.50
In a relationship and living together	45	15	0.64	0.16–2.59
In a relationship and not living together	476	40	1.73	0.79–3.79
*Household income level*				
I do not get an income	69	15	1.75	0.38–8.13
R1-1600	260	22	2.18	0.75–6.33
[R1601+]	263	37	1.52	0.65–3.54

Data source: AYAZAZI RIGHTS survey focusing on sexual and reproductive health among adolescents in South Africa.

Analyses were adjusted for age, race, relationship status, sexual orientation, frequency of alcohol consumption, having children, being in school or employed, HIV status, average monthly household income, number of adults in household, number of children in household, number of seniors in household, not able to cope during COVID-19, being upset during COVID-19, anxiety during COVID-19, and worrying during COVID-19

* AYAZAZI*  Understanding HIV Risk Among Youth in South Africa, *CI *confidence interval, *Events* number of participants reporting IPV perpetration, *IPV* intimate partner violence, *N* number of participants included in analysis, *OR* odds ratio,  *RIGHTS *Rapid Investigation of Gendered Health outcomes in the Time of SARS-CoV-2

In our PS-MW weighted sample, the estimated odds of IPV perpetration were 57% higher (CI 15% lower to 192% higher) among men reporting economic hardship than those not reporting economic hardship. Economic hardship elevated the odds of perpetrating sexual IPV by 350% (CI 16–1600%), with very large variation over the CI, although greater precision than in the main analysis. The point estimate of economic hardship’s hypothesized effect on physical IPV was a 26% increase in the odds, with substantial imprecision in terms of the direction and magnitude of the effect (CI 35% lower to 163% higher odds), although again more precise than the estimate for sexual IPV perpetration.

## Discussion

We estimated that, among young South African men, living in urban settings, the effect of economic hardship was an 83% elevation in the odds of perpetrating IPV. A sevenfold increased odds of sexual IPV by economic hardship was observed, while evidence was unclear for an effect on physical IPV by economic hardship. These findings were supported by a propensity scores analysis that balanced covariates through matching weights. Although the point estimates were substantially attenuated, tighter confidence intervals improved their precision. Economic hardship consistently influenced sexual IPV perpetration risk in both our main analysis and PS-MW sensitivity analysis. Our effect modification analyses did not observe a linear trend in the OR of economic hardship for overall IPV perpetration over the income strata. Contrary to our hypothesis about effect modification by relationship status, our findings suggest that single men had higher odds of IPV perpetration compared to men in relationships; however, this is likely an artifact of  due to reverse causality due to cross-sectional measurement of exposure and outcome.

Our findings may be explained by the gender role strain paradigm, which posits that IPV perpetration by men can be triggered by economic hardship, which threatens perceived masculine gender roles, potentially causing a shift toward more violent forms of masculinity (Closson et al. 2020; Pleck 1995). In a study of economic stress during COVID-19, IPV perpetration worsened with the decline of men’s socioeconomic status, more so if they felt their position was threatened already, and particularly when the woman was working (Arenas-Arroyo et al. 2021). IPV perpetration causal mechanisms have also been described with structural equation modeling in South Africa, evaluating multiple predictors and pathways between hegemonic masculinity and other potential underlying mechanisms (Gibbs et al. 2018), although these are difficult to compare with ours duowinge to the differences in methodological approaches.

### Public health interventions

More equitable gender norms between partners can reduce sexual entitlement and improve mutuality in deciding to engage in sexual intercourse (Hatcher et al. 2014; Beckwith et al. 2022). Some authors have recommended that interventions aimed at reducing IPV should be comprehensive enough to address risky sexual behaviors and alcohol abuse while also focusing on gender norms and power dynamics in intimate partner relationships (Mthembu et al. 2016; Russell et al. 2014). Several interventions that have been designed to address the social determinants of violence in South Africa include Thula Sana, the Sinovuyo Caring Families Programme, PREPARE, Skhokho Supporting Success, Stepping Stones, Stepping Stones and Creating Futures and IMAGE (Shai and Sikweyiya 2015). These interventions make use of prevention strategies which were designed to include theory of change, cultural relevance, participatory methods and evaluation through randomized controlled trials (Shai and Sikweyiya 2015). Evaluations of these interventions show that they have led to positive outcomes such as increased positive parenting with less violent or abusive discipline, significant reductions in IPV among teenagers, reduced cases of reported risk behaviors and IPV in men and significant reductions in women’s experiences of sexual IPV (Shai and Sikweyiya 2015).

Community and group-level gender transformative and microfinance interventions have been designed and tested to prevent IPV and a host of gender inequities, unhealthy relationship dynamics and attitudes, as well as mental health and HIV. Two notable randomized controlled trials (RCTs) in South Africa were designed to address financial, sociocultural, and psychological risk and contributing factors for IPV: Stepping Stones and Creating Futures and Intervention with microfinance for AIDS and Gender Equity (IMAGE) Study (Shai and Sikweyiya 2015).

Combining the Stepping Stones group intervention with the Creating Futures economic empowerment program aimed to address violence among young men and women (Jewkes et al. 2014). The intervention targeted young people (aged 18–30) living in informal settlements and did peer-facilitated group sessions for livelihood strengthening. This was a quasi-experimental study with a one-year follow-up and findings showed a significant reduction in women’s experience of sexual IPV in the prior 3 months, an improvement in gender attitudes among both men and women, and an increase in more equitable relationships at 12 months of follow-up (Jewkes et al. 2014). The findings suggest a positive impact on economic empowerment and attitudes but a mixed effect on violence reduction.

Similarly, the IMAGE study was designed to enhance economic well-being, social capital, and empowerment to decrease vulnerability to IPV and HIV by combining microfinance with gender and HIV structured training and community mobilization for women (aged 14–35) living in poverty in Limpopo, South Africa (Pronyk et al. 2006). Matched villages were randomized to intervention or control, with two-year follow-up questionnaires to assess IPV and gender equity outcomes. In the intervention group, intimate partner violence decreased by 55%, demonstrating the effectiveness of the combined intervention. The findings further suggest that integrated microfinance and training interventions can reduce intimate partner violence and influence risk environments for HIV in southern Africa (Pronyk et al. 2006).

The South African government introduced social assistance measures during the pandemic such as unemployment grants and food aid, which were expected to provide support to 8 million people (Villiers et al. 2020; Bhorat et al. 2021; Abdool Karim 2020). However, these efforts only reached a third of South African citizens (Moosa et al. 2021). While government led structural and social relief measures needed strengthening to mitigate economic hardships during the pandemic, more efforts could have been put toward psychosocial support (Mahlangu et al. 2022).

Randomized controlled trials (RCTs) of the interventions above were meta-analyzed (Leight et al. 2023; Allan-Blitz et al. 2023), finding a pooled reduction of 22% (CI 3–37%) in the odds of past year experiences of IPV by women through group-based or community-based programming. A meta-analysis of microfinance RCTs found a 13% reduction of psychological and emotional IPV (CI 5–20%), a 24% drop in sexual violence (CI 10–37%), an 18% decline in controlling behaviors (CI 8–26%), and insufficient evidence to interpret an effect on physical IPV perpetration (SMD, 0.89; 95% CI 0.76–1.04) (Allan-Blitz et al. 2023).

### Limitations

This study has several limitations. First, as a cross-sectional study, there is the possibility of reverse causality whereby people who perpetrated IPV may have been more likely to experience economic hardship, which cannot be ruled out with this data. Second, the sampling was based on convenience sampling which may limit the generalizability of our findings. Third, in terms of information bias, there was potential for social desirability bias, which may have influenced the reporting of factors such as IPV perpetration, exposure to economic hardship, and covariate values. However, we have no reason to believe the likelihood of outcome misclassification would differ between exposure groups, and thus would only bias our results toward the null, leading to potentially conservative estimates.

Fourth, we were unable to separate effects of economic hardship from lockdown effects on IPV perpetration risk with our data. However, others have noted that economic hardship is more predictive of IPV perpetration than lockdown (Arenas-Arroyo et al. 2021). Fifth, we could not measure all known confounders, particularly childhood trauma, community violence, and substance use disorder, leaving residual confounding to the extent these potential confounders were not proximally adjusted for through our measured covariates although the extent and direction of residual confounding required to completely explain away our main analysis effect estimate would be a risk ratio relationship of 3.06 between the residual confounding and both economic hardship and perpetration of IPV (VanderWeele and Ding 2017; Mathur et al. 2018).

### Future research

Unpacking the strong association of economic hardship with sexual IPV perpetration is warranted. Testing our conclusions in other populations, settings, and with different data sources, especially using longitudinal designs, would improve our understanding of causal mechanisms in IPV perpetration by economic hardship in this youth subpopulation. Separating effects of economic hardship from lockdown is also a task requiring investigation given the relevance to pandemic policy and decision-making, given the weaker association between lockdown and either sexual or physical IPV as compared with economic hardship (Arenas-Arroyo et al. 2021). Qualitative research with participants experiencing economic hardship and perpetrating IPV could inform the design of prospective research into IPV perpetration, describing potential mechanisms that could be assessed quantitatively.

### Conclusion

Our findings confirm previous research indicating that economic hardship could be an important trigger for IPV perpetration by young men. Group and community-level efforts, such as gender transformative training, community mobilization, and microfinancinge, might be adaptedtailored to young men experiencing economic hardship.

## Data Availability

Study data may be made available from the corresponding author upon reasonable request. Analysis code may be used freely with citation: https://github.com/CAndrewBasham/Economic_Hardship_IPV_perpetration/
